# Discovery of a selective, safe and novel anti-malarial compound with activity against chloroquine resistant strain of *Plasmodium falciparum*

**DOI:** 10.1038/srep13838

**Published:** 2015-09-08

**Authors:** Ankita Agarwal, Sarvesh Paliwal, Ruchi Mishra, Swapnil Sharma, Anil Kumar Dwivedi, Renu Tripathi, Sarika Gunjan

**Affiliations:** 1 Department of Pharmacy, Banasthali University, Rajasthan, 304022, India; 2 Pharmaceutics Division, CSIR- Central Drug Research Institute (CSIR-CDRI), Sector 10, Jankipuram Extension, Sitapur Road, Lucknow, 226031, India; 3 Parasitology Division, CSIR-Central Drug Research Institute (CSIR-CDRI), Sector 10, Jankipuram Extension, Sitapur Road, Lucknow, 226031, India

## Abstract

In recent years the DNA minor groove has attracted much attention for the development of anti-malarial agents. In view of this we have attempted to discover novel DNA minor groove binders through *in-silico* and *in-vitro* workflow. A rigorously validated pharmacophore model comprising of two positive ionizable (PI), one hydrophobic (HY) and one ring aromatic (RA) features was used to mine NCI chemical compound database. This led to retrieval of many hits which were screened on the basis of estimated activity, fit value and Lipinski’s violation. Finally two compounds NSC639017 and NSC371488 were evaluated for their *in-vitro* anti-malarial activities against *Plasmodium falciparum* 3D7 (CQ sensitive) and K1 (CQ resistant) strains by SYBR green-I based fluorescence assay. The results revealed that out of two, NSC639017 posses excellent anti-malarial activity particularly against chloroquine resistant strain and moreover NSC639017 also appeared to be safe (CC_50_ 126.04 μg/ml) and selective during cytotoxicity evaluation.

Malaria is one of the world’s most devastating infectious diseases in terms of both mortality and morbidity. There are about 0.5 billion clinical attacks every year, including 2–3 million severe attacks, with 0.8 to 1.2 million deaths annually^1^. The situation is rapidly worsening, mainly due to development of resistance to the existing first line drugs, such as chloroquine and pyrimethamine^2^. Hence, chloroquine, which has been the most common anti-malarial drug for decades, is now practically ineffective and emergence of resistance to other drugs such as mefloquine, halofantrine, or artemisinin is beginning to appear^3^. For those reasons, WHO now recommends the use of artemisinin-based combination therapy (e.g., artesunate/mefloquine, artesunate/amodiaquine) in order to delay the development of resistant strains^4^. The need for safe and affordable antiplasmodial therapies capable of overcoming the problems of parasite resistance makes the identification of new drug candidates an urgent priority.

Among available malarial targets, DNA minor groove is considered as a better option for the development of anti-malarial drugs and the proof of concept has come from discovery of broad antiparasitic including anti-malarial activity of pentamidine and other diamidines which exerts their action by binding to DNA minor groove. Undoubtedly this has created interest for development of antiplasmodial compounds targeting DNA minor groove^5^. In view of this we have made an effort to implement *in-silico* protocols in association with wet lab experimentation to identify novel and safe *Pf* DNA minor groove binders with ability to act particularly against resistant strain of *P. falciparum*.

## Results and Discussion

### Pharmacophore generation and statistical evaluation

Pharmacophore models with varying number and type of features were generated and evaluated on the basis of cost fuction calculated in bits by HypoGen module^6^. The chosen pharmacophore model comprising of two positive ionizable (PI), one hydrophobic (HY) and one ring aromatic (RA) features exhibited a fixed cost value of 135.37 bits, well separated from the null cost (247.99 bits). The model also showed a difference of 98.79 bits between total cost (149.20) and the null cost. For a significant model the value of configuration should be less than 17 and the model exhibited a value of 9.80 with RMS value of 0.84 and squared correlation coefficient value of 0.86 confirming the significance of the model. Supplementary Figure S1 shows the graph plotted between the actual and estimated activities of the training set of compounds, as predicted by the model.

### Pharmacophore mapping

**Figure 1:**
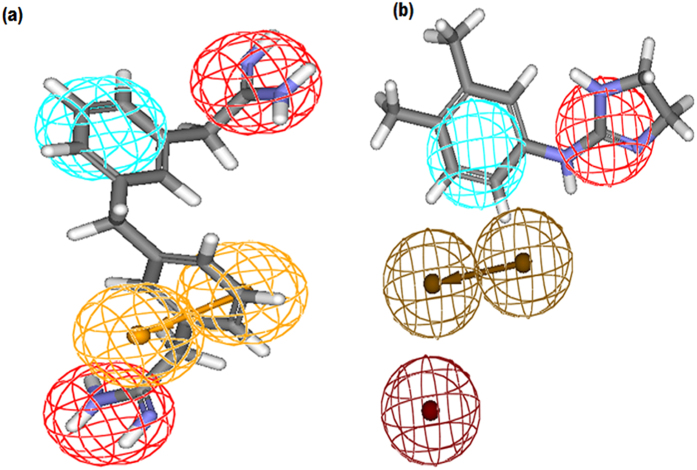
(**a**) Pharmacophore mapping of the most active training set compound 13a, (**b**) Pharmacophore mapping of the least active training set compound 36b.

During mapping of training set compounds it was observed that most active compound 13a (IC_50_ 0.002 μM) of training set mapped well over the model with a fit value of 8.027. Both the positive ionizable (PI) features mapped over the two guanidine moieties present at the terminal position. One ring aromatic (RA) feature mapped on to the benzene ring while hydrophobic feature mapped on to another benzene ring (Fig. 1a). On the other hand least active compound 36b (IC_50_ 17.1 μM) with a poor fit value of 4.671 clearly missed one RA and PI features during mapping (Fig. 1b).

### Validation of pharmacophore model

In order to evaluate the statistical quality, prognostic nature, and overall fitness of the pharmacophore model, rigorous validation was carried out using CatScramble test, internal and external test set prediction and Güner-Henry scoring method.

### CatScramble validation

During the validation process random spreadsheets were generated using the same training set compounds and features as used during the generation of original pharmacophore model. A total of 99 random spreadsheets were generated to achieve a confidence level of 99%. The results of the randomized data were analyzed and it was observed that the statistics of selected model is far better as compared to the 99 randomly generated models (Supplementary Figure S2). The results of CatScramble clearly shows that the generation of original model is not by chance rather it is an outcome of true correlation between structures of training set compounds and *Pf* DNA minor groove inhibitory activity.

### Internal test set validation

**Figure 2:**
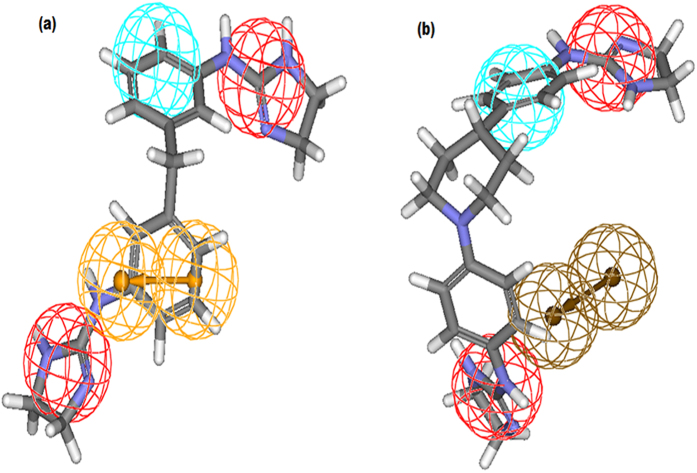
(**a**) Pharmacophore mapping of the most active test set compound 13b, (**b**) Pharmacophore mapping of the least active test set compound 23a.

Activity prediction and pharmacophore mapping of 23 test set compounds was carried out with an objective to verify whether generated pharmacophore model is capable of predicting the activities of compounds not included in training set and classifying them correctly as actives or inactives. A squared correlation coefficient value of 0.81 (Supplementary Figure S1) between actual and estimated activities of the test set clearly demonstrated good prediction ability of the pharmacophore model. During mapping of the test set compounds it was observed that most active compound 13b (Fig. 2a) mapped all four features of the pharmacopore with a fit value of 7.53 (IC_50_ 0.012 μM), whereas least active compound 23a (IC_50_ 10.4 μM) showed a fit value of 5.36 and missed one PI feature (Fig. 2b).

### External test set validation

For the assurance of applicability, predictivity and soundness of the model an external dataset of 30 structurally diverse compounds with known *Pf* DNA minor groove inhibitory activity were mapped on to the pharmacophore model and an comparison between estimated the actual activity was made (Supplementary Table S1). Observed predictive r^2^ value of 0.71 clearly provides a reflection of the predictivity and soundness of the chosen pharmacophore model.

### Güner-Henry (GH) scoring method

In order to ascertain the performance of the pharmacophore model during virtual screening the following measures were critically analyzed; hit list (Ht), number of active percent of yields (%Y), percent ratio of actives in the hit list (%A), enrichment factor (E), false negatives, false positives, and goodness of hit score (GH scoring method). It is apparent from the results of GH scoring method (Table 1) that the chosen model succeeded in retrieving 84% of the active compounds, 5 inactive compounds (false positives), and predicted 2 active compounds as inactive (false negatives). A GH score of 0.71 clearly indicates the high quality of the model.

### Database screening

Utility of any pharmacophore model lies in its ability to virtually screen large chemical compound databases and since the developed pharmacophore model showed all the signs of its soundness and universality, it was used to screen National Cancer Institute Database. The retrieved hits were screened on the basis of their fit and estimated value which led to retention of 9 out of 167 hits (NSC 639017, NSC 371488, NSC 690245, NSC 371487, NSC 690246, NSC 295561, NSC 685847, NSC 318794 and NSC 212196) with estimated activity of 0.001, 0.001, 0.002, 0.002, 0.003, 0.004, 0.006, 0.008 and 0.009 μM and fit value of 8.59, 8.35, 7.90, 7.67, 7.49, 7.14, 7.02, 6.87 and 6.52 recpectively. Since NSC639017 and NSC371488 exhibited best fit and estimated value they were subjected to Tanimoto similarity measure and wet lab experimentation. The structures of identified hits along with their estimated activity, fit value, chemical structure and mapping pattern are given in Table 2.

### Tanimoto similarity measure

Before proceeding to experimental validation the identified hits were checked for novelty by comparing their similarity with known DNA minor groove binders from binding database. NSC639017 and NSC371488 showed low tanimto similarity indices of 0.13 and 0.13 to all the structures of established *Pf* DNA minor groove binders, firmly establishing their novelty.

### *In-vitro* anti-malarial assay

Since NSC639017 and 371488 exhibited good estimated activity, fit value and low tanimoto similarity score, they were procured from National Cancer Institute, USA and subjected to experimental validation using an *in-vitro* anti-malarial assay performed against *Plasmodium falciparum* 3D7 (CQ-sensitive) and K1 (CQ resistant) strains.

The fifty percent inhibitory concentration (IC_50_) was determined for NSC639017, NSC371488 and standard respectively using fluorescence reader. The results (Table 3) of anti-malarial screening revealed that NSC639017 posses excellent anti-malarial agent with IC_50_ value of 1.15 ± 0.2 μg/ml against *Pf*3D7 strain and 0.38 ± 0.02 μg/ml against *Pf*K1 strain when compared to standard chloroquine with IC_50_ value of 0.005 μg/ml for *Pf*3D7 strain and 0.598 μg/ml for *Pf*K1 strain. The second hit NSC371488 showed moderate activity with IC_50_ value of 3.08 ± 0.02 μg/ml against *Pf*3D7 strain and 1.54 ± 0.03 μg/ml in case of *Pf*K1 strain. It is noticeable that the results of *in-vitro* anti-malarial assay are in line to pharmacophore mapping results where NSC639017 exhibited a higher fit value of 8.59 in comparison to NSC371488 which showed a fit value of 8.35.

### *In-vitro* cell cytotoxicity assay

With an aim to establish the safety of the identified hits they were subjected to cytotoxicity evaluation using mammalian VERO cell line. NSC639017 with CC_50_ value of 126.04 μg/ml appeared to be safe than NSC371488 with CC_50_ value of 36.89 μg/ml. Moreover the calculated selectivity indices (CC_50_/IC_50_) have shown that the NSC639017 has better selectivity index value of 109.6 for *Pf*3D7 strain and 331.68 for *Pf*K1 strain (Table 2) when compared to NSC371488 with selectivity index of 11.97 for *Pf*3D7 strain and 23.95 for *Pf*K1 strain.

In conclusion, through our pharmacophore based virtual screening workflow we have identified a novel and structurally diverse DNA minor groove binder with good anti-malarial activity against CQ resistant strain of *Plasmodium*, the safety and selectivity of NSC639017 makes it an ideal candidate for the further development as anti-malarial agent.

## Methods

### Data set preparation and conformational analysis

A compound collection of 61 minor groove binders with wide range of activity (0.0023 to 17.1 μM) and structural diversity (Supplementary Table S2) were used for development of pharmacophore model^7^. Energy of all the compounds were minimized using CHARMm force field^8^. A maximum of 255 diverse conformers were generated for each molecule using best flexible conformation generation module^9^ of Accelrys Discovery studio v2.0. In total 38 compounds were used as training set while the rest of 23 compounds were used as internal test set to validate the pharmacophore model.

### Pharmacophore generation and statistical evaluation

Chemical features such as hydrogen bond acceptor (HBA), hydrogen bond donor (HBD), hydrophobic (HY), positive ionizable (PI) and ring aromatic (RA) were used to generate various pharmacophore models keeping minimum and maximum features value between 0 and 5. During the course of modeling, it was observed that two PI, one HY and one RA feature are important and are appearing in most of the useful models. Hence these features, were used to generate a final set of ten pharmacophore models, with default uncertainty value of three, which means that the actual activity of a particular inhibitor is supposed to be situated somewhere in an interval ranging from one-third to three times the reported bioactivity value of that inhibitor^10^,^11^. Out of the chosen set of ten pharmacophore models, best model was selected on the basis of cost difference (null-total), fixed cost, RMS values (root mean square values), correlation coefficient and configuration cost^12^. In addition to aforesaid quality parameters, the soundness and predictability of the generated pharmacophore model was also judged using Fisher randomization test, internal and external test set prediction and GH scoring.

### CatScramble validation

The CatScramble statistical validation technique based on Fischer’s randomization test has been used to check whether there is a strong correlation between the chemical structures and the biological activity under investigation. The statistical significance has been calculated using the following equation.


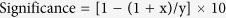


where x = total number of hypotheses having a total cost lower than best significant hypothesis and y = number (HypoGen runs initial + random runs). To obtain a 99% confidence level, 99 random spreadsheets were generated (y = 20) and every generated spreadsheet was submitted to HypoGen using the same experimental conditions (functions and parameters) as the initial run. The statistical output of the generated models was analyzed^13^.

### Internal test set validation

An internal test set containing 23 compounds, representing diverse activity classes and different functional groups was employed to assess the predictive power of the developed model. All the molecules of test set were mapped onto the generated pharmacophore model, and thus, the prediction of desired activity was made. The activity prediction of the test set compounds was measured in terms of the squared correlation coefficient (r^2^)^14^.

### External test set validation

The ultimate objective behind the pharmacophore modeling is to use them in the lead identification and optimization phases of the drug discovery paradigm. However, prediction ability of the developed model (s) should be well assessed before its use. External test set validation is one of the best ways to assess the prediction capability and universality of the developed model^15^. In the present study, the developed pharmacophore was validated using a structurally diverse external test set comprising of 30 *Pf* DNA minor groove binders^16^. The prediction capability of the chosen pharmacophore model was also evaluated on the basis of squared correlation coefficient (r^2^).

### Güner-Henry (GH) scoring method

In order to assess the precision of the pharmacophore the GH scoring method was used. The appropriate use of this method in a drug discovery process improves the ability to identify and optimize hits and confirm their potential to serve as scaffolds for producing new therapeutic agents. 290 structurally diverse known *Pf* DNA minor groove binders were selected from eleven publications^16^,^17^,^18^,^19^,^20^,^21^,^22^,^23^,^24^,^25^,^26^. The method comprises of computing the following: the percent yield of actives in a database (%Y, recall), the percent ratio of actives in the hit list (% A, precision), the enrichment factor E and the GH score. The GH score ranges from 0 to 1, where a value of 1 signifies the ideal pharmacophore model. The aforementioned measures were computed using the equation 1–4^27^,^28^.


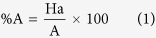



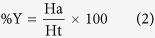



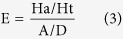



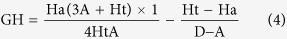


%A is the percentage of known active compounds retrieved from the database (precision); Ha, the number of actives in the hit list (true positives); A, the number of active compounds in the database; %Y, the percentage of known actives in the hit list (recall); Ht, the number of hits retrieved; D, the number of compounds in the database; E, the enrichment of active compounds in the virtual screening hit list in comparison to the non-filtered database and GH is the Güner-Henry score.

### Database screening

Virtual screening has been widely used for lead identification in drug discovery programs. Virtual screening methods are generally divided into ligand-based virtual screening and structure-based virtual screening. Pharmacophore-based database searching is considered a type of ligand-based virtual screening, which can be efficiently used to find novel, potential leads for further development from a virtual database. A rigorously validated pharmacophore model includes the chemical functionalities responsible for bioactivities of potential drugs, therefore, it can be used to perform a database search by serving as a 3D query. Validated pharmacophore model containing required pharmacophore features, shapes and excluded volumes was used to mine the NCI 3D database to identify new molecules which share its features and can thus exhibits the desired biological response. The NCI Open Database contains 265,000 freely available structures with 3D coordinates, some ADME proprieties and information about predicted activity along with a restricted collection of 2,500 compounds with some lead-like properties such as 5 or fewer rotatable bonds, 1 or less chiral centers and pharmacologically desirable features (i.e., they are not electrophilic, unstable, organometallic, polycyclic aromatic hydrocarbons, etc). The retrieved hits were rigorously screened on the basis of fit value, estimated value and Lipinski’s rule-of-five^29^.

### Tanimoto similarity measure

The novelty of retrieved hits was checked by employing pair-wise tanimoto similarity indices protocol^30^. Ideally Tanimoto similarity score should not exceeds a 0.99 threshold value.

### *In-vitro* anti-malarial assay

The anti-malarial activity was evaluated *in-vitro* against *P. falciparum* 3D7 (CQ-sensitive) and K1 (CQ resistant) strains by SYBR green-I based fluorescence assay^31^. The chloroquine sensitive (*Pf*3D7) and chloroquine resistant (*Pf*K1) strains of *P. falciparum* were cultured in RPMI-1640 (HEPES modified) medium (Sigma) supplemented with 0.5% AlbuMaxII, 0.2% glucose, 0.2% NaHCO_3_ and 15 μM hypoxanthine according to the method of Trager and Jensen^32^. Parasite growth rate and stages were determined by the examination of Giemsa’s stained thin smears of the RBCs. Two fold serial dilutions of lead compound and chloroquine (standard anti-malarial) were prepared in 96 well plates and then 50 μl asynchronous culture of infected erythrocytes with 1–1.5% parasitaemia and 2–3% haematocrit was added to each well (100 μl-final volume). Eight wells were treated as positive control (with parasite, without drug) and 4 wells as negative controls (without parasite and drug). Plates were incubated in CO_2_ incubator maintained at 37 °C for 72 h. After 72 h, 100 μl lytic buffer containing SYBR Green 1X final concentration was added to each well and incubated for 1–2 h at room temperature in dark. Plates were read under fluorescence reader at Ex. 485 nm, Em. 535 nm. Assessment of anti-malarial activity of compounds towards *P. falciparum* was made on the basis of fifty percent inhibitory concentration values (IC_50_)^33^ determined on the basis of DNA content of the parasite.

### *In-vitro* cell cytotoxicity assay

The cytotoxicity evaluation of active compound was made on VERO cell line (Monkey kidney cell line) as per earlier reported protocol^34^. The monkey kidney cell line (VERO) were maintained *in-vitro* in MEM medium supplied with 15% Foetal Bovine Serum (FBS) and 5% CO_2_ at 37 °C. An appropriate serial dilution was prepared in culture plates and the cells were exposed to chosen concentrations of test and standard compound for three days, 10% of cell viability marker resazurin was added and read under fluorescent reader at Ex. 530 ± 25 nm and Em. 590 ± 25 nm for calculation of the median cytotoxic concentration (CC_50_). The selective index (SI) was calculated by using the formula CC_50_/ IC_50._

## Additional Information

**How to cite this article**: Agarwal, A. *et al*. Discovery of a selective, safe and novel anti-malarial compound with activity against chloroquine resistant strain of *Plasmodium falciparum*. *Sci. Rep*. **5**, 13838; doi: 10.1038/srep13838 (2015).

## Supplementary Material

Supplementary Information

## Figures and Tables

**Table 1:** Pharmacophore model evaluation based on the Güner-Henry scoring method.

Serial No.	Parameter	*Pf* DNA minor groove binders
1	Total molecules in database (D)	381
2	Total Number of active in database (A)	29
3	Total Hits (Ht)	32
4	Active Hits (Ha)	27
5	% Yield of actives [(Ha/Ht) × 100]	84.37
6	% Ratio of actives [(Ha/A) × 100]	93.10
5	Enrichment factor (E) [(Ha × D)/(Ht × A)]	11.08
6	True positives (Active Hits) [Ha]	27
7	True negatives	352
8	False negatives [A-Ha]	2
9	False positives [Ht-Ha]	5
10	Goodness of hit score	0.71

**Table 2:** Hits retrieved from NCI database search along with their estimated activity, fit value, chemical structure and mapping on the developed pharmacophore.

Name of hits	Estimated activity IC_50_ (μM)	Fit value	Chemical structure	Mapping of hits on pharmacophore
NSC639017	0.001	8.59	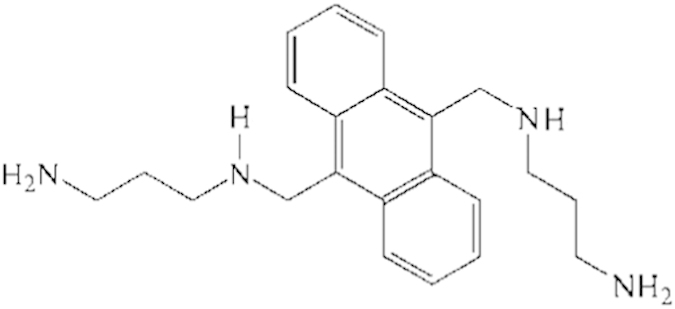	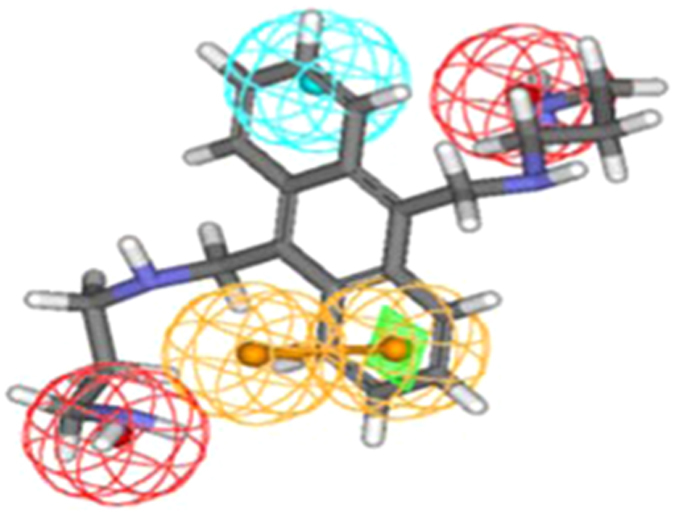
NSC371488	0.001	8.35	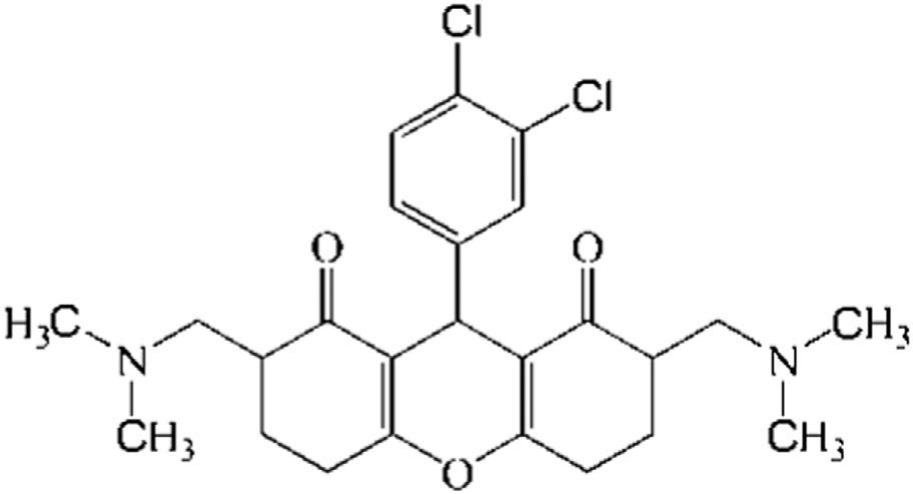	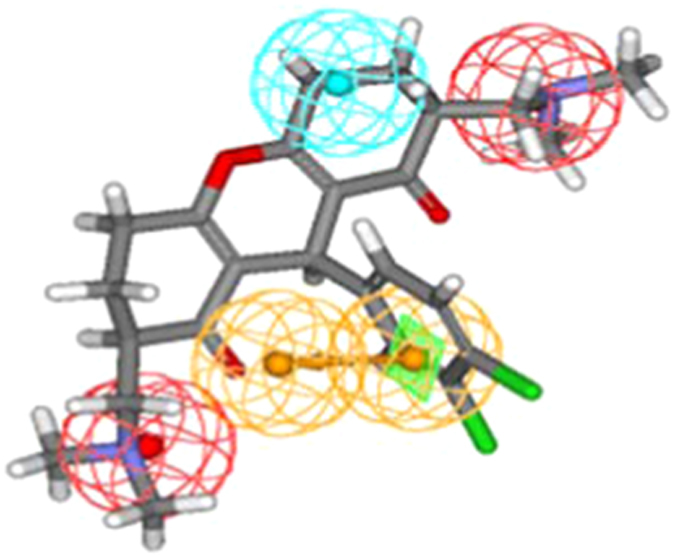

**Table 3:** Anti-malarial efficacy of identified hits against *Plasmodium falciparum* and safety index against VERO cell line.

Name of hits	IC_50_ (μg/ml)		CC_50_ (μg/ml)(VERO Cellline)	SI(CC_50_/IC_50_)	
	*Pf*3D7 (CQsensitive)	*Pf*K1 (CQresistant)		*Pf*3D7	*Pf*K1
NSC639017	1.15 ± 0.2	0.38 ± 0.02	126.04	109.6	331.68
NSC371488	3.08 ± 0.02	1.54 ± 0.03	36.89	11.97	23.95
Chloroquine	0.005	0.598	353.29	70600.00	590.78
