# Supplementary material for: Discovery of a selective, safe and novel anti-malarial compound with activity against chloroquine resistant strain of Plasmodium falciparum
Source: Sci Rep. 2015 Sep 8;5:13838. doi: 10.1038/srep13838 (PMC4561909; doi:10.1038/srep13838)
Supplement: Supplementary Information [file srep13838-s1.pdf]

Discovery of a selective, safe and novel anti-malarial compound with activity against chloroquine resistant strain of *Plasmodium falciparum*

Ankita Agarwal[1], Sarvesh Paliwal[1*], Ruchi Mishra[1], Swapnil Sharma[1], Anil Kumar Dwivedi[2], Renu Tripathi[3] & Sarika Gunjan[3]

Table S1. Actual and estimated Activity values of the thirty external test set compounds

Compounds Name	Actual Activity	Estimated Activity	Actual Activity (-log value)	Estimated Activity (-log value)
2	0.118	0.033	0.928	1.481
3	0.365	0.032	0.437	1.494
5	0.125	0.032	0.903	1.494
8	0.106	0.032	0.974	1.494
11	0.739	0.034	0.131	1.468
12	0.325	0.035	0.488	1.455
14	0.229	0.032	0.640	1.494
15	0.052	0.035	1.283	1.455
17	0.038	0.039	1.420	1.408
18	0.084	0.048	1.075	1.318
20	0.055	0.039	1.259	1.408
21	0.058	0.046	1.236	1.337
23	0.026	0.051	1.585	1.292
25	0.175	0.056	0.756	1.251
27	0.159	0.044	0.798	1.356
29	0.028	0.046	1.552	1.337
32	0.043	0.046	1.366	1.337
33	0.095	0.043	1.022	1.366

35	0.195	0.051	0.709	1.292
39	0.064	0.038	1.193	1.420
46	0.002	0.329	2.698	0.482
47	0.005	0.292	2.301	0.534
48	0.002	1.553	2.698	-0.191
49	0.009	0.209	2.045	0.679
52	0.002	0.219	2.698	0.659
53	0.013	0.266	1.886	0.575
54	0.011	0.415	1.958	0.381
57	0.041	0.09	1.387	1.045
58	0.002	0.16	2.698	0.795
59	0.007	0.198	2.154	0.703

Table S2. Structure and biological activity of Guanidine and 2-aminoimidazoline analogues as *P. falciparum* DNA minor groove binders

**Diphenyl dicationic compounds**				
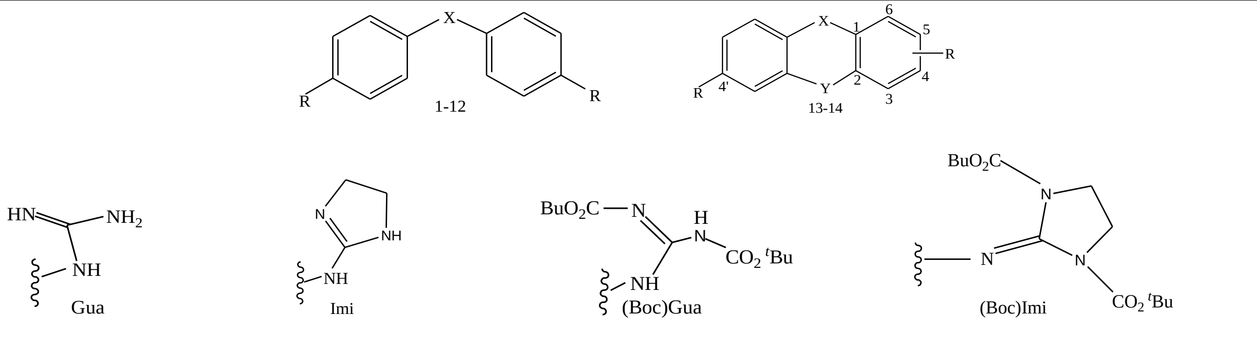				
**Compounds Name**	**R**	**X**	**Y**	**IC_{50} Values (μM)**
**1a**	Gua	NH	-	0.018

**1b**	Imi	NH	-	0.0088
**1c**	$(EtO)_2CHCH_2$-Gua	NH	-	0.113
**1d**	(Boc)Gua	NH	-	0.077
**1e**	(Boc)Imi	NH	-	0.059
**2a**	Gua	CH_2	-	0.032
**2b**	Imi	CH_2	-	0.0157
**2c**	$(EtO)_2CHCH_2$-Gua	CH_2	-	0.036
**3a**	Gua	O	-	0.046
**3b**	Imi	O	-	0.038
**4a**	Gua	S	-	0.035
**4b**	Imi	S	-	0.025
**5a**	Gua	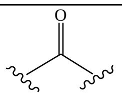	-	0.068
**5b**	Imi	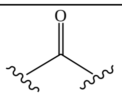	-	0.129
**6a**	Gua	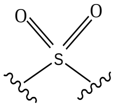	-	0.444
**6b**	Imi	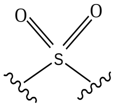	-	5.6
**6e**	(Boc)Imi	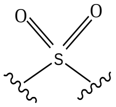	-	0.055
**7a**	Gua	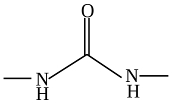	-	0.096

**7b**	Imi	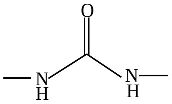	-	0.028
**8a**	Gua	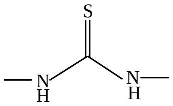	-	0.607
**9a**	Gua	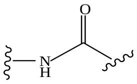	-	0.055
**9b**	Imi	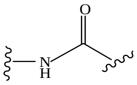	-	0.028
**10a**	Gua	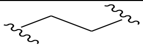	-	0.019
**10b**	Imi	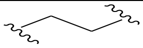	-	0.016
**11a**	Gua	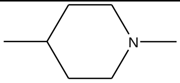	-	0.041
**11b**	Imi	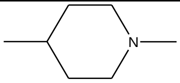	-	0.011
**12a**	Gua	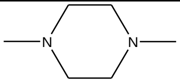	-	0.0152
**12b**	Imi	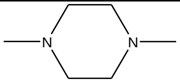	-	0.0123
**13a**	4,4'-Gua	-	CH_2	0.0023
**13b**	4,4'-Imi	-	CH_2	0.0115
**14a**	5,4'-Gua	CH_2	CH_2	0.0088
**14b**	5,4'-Imi	CH_2	CH_2	0.0186
**Diphenyl monocationic compounds**				
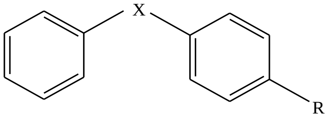				

Compounds Name	R	X	IC_{50} Values (µM)
**15a**	Gua	NH	1.6
**15b**	Imi	NH	0.549
**16a**	Gua	CH_2	3.8
**16b**	Imi	CH_2	1.7
**17a**	Gua	O	7.9
**17b**	Imi	O	5.7
**18b**	Gua	S	1.2
**19b**	Imi	CO	5.2
**20a**	Gua	NH-CO-NH	4.1
**20b**	Imi	NH-CO-NH	3.4
**21a**	Gua	NH-CS-NH	8.7
**21b**	Imi	NH-CS-NH	3.1
**22a**	Gua	NH-CO	13.3
**23a**	Imi	CO-NH	10.4

Phenyl monocationic compounds

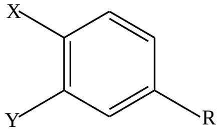

Compounds Name	R	X	Y	IC_{50} Values (µM)
**25b**	Imi	NH_2	H	15.0

**26a**	Gua	Et_2NH	H	5.7
**26b**	Imi	Et_2NH	H	15.0
**28a**	Gua	Et	H	9.0
**29a**	Gua	CH_3S	H	1.3
**29b**	Imi	CH_3S	H	1.7
**30b**	Imi	CH_3CO	H	5.7
**31a**	Gua	4-piperidin-1-yl	H	7.9
**31b**	Imi	4-piperidin-1-yl	H	12.0
**32a**	Gua	fused cyclopentane	-	0.95
**32b**	Imi	fused cyclopentane	-	5.3
**33a**	Gua	Fused 1,4-dioxane	-	9.0
**35b**	Imi	CH_3O	CH_3O	14.8
**36a**	Gua	CH_3	CH_3	12.2
**36b**	Imi	CH_3	CH_3	17.1

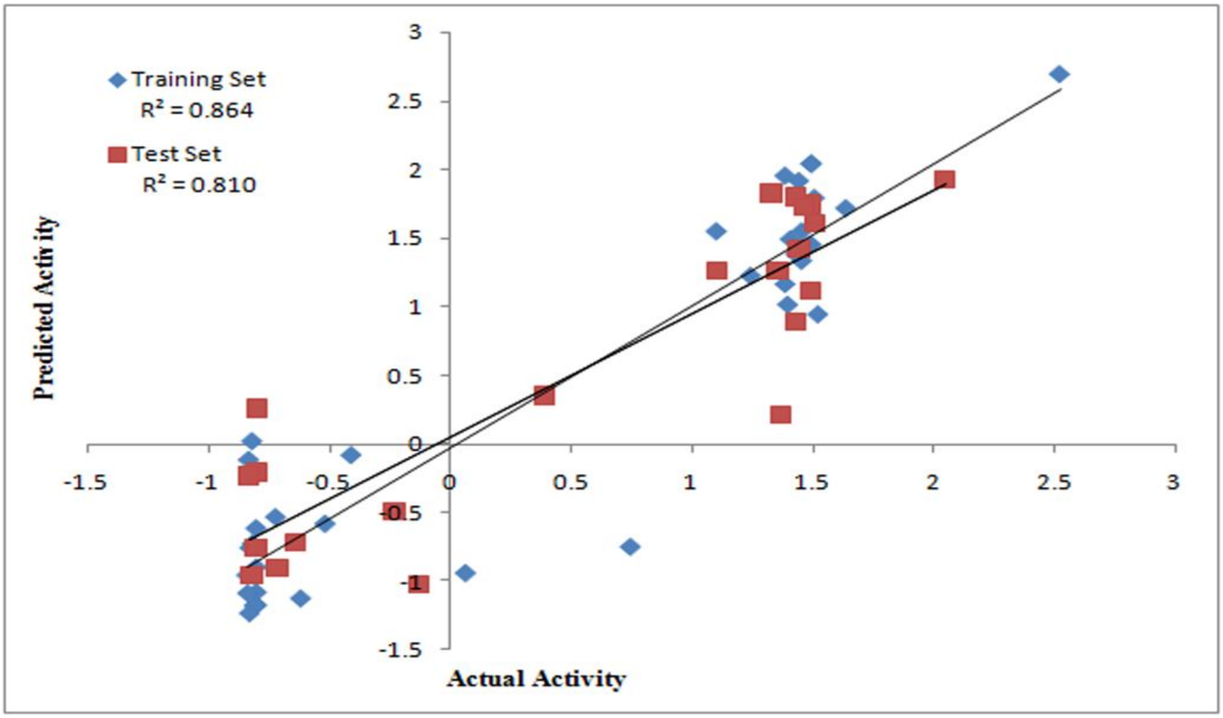

Figure S1. Graph plotted between actual and predicted value for *P. falciparum* DNA minor groove binders using pharmacophore analysis for training and test set of compounds.

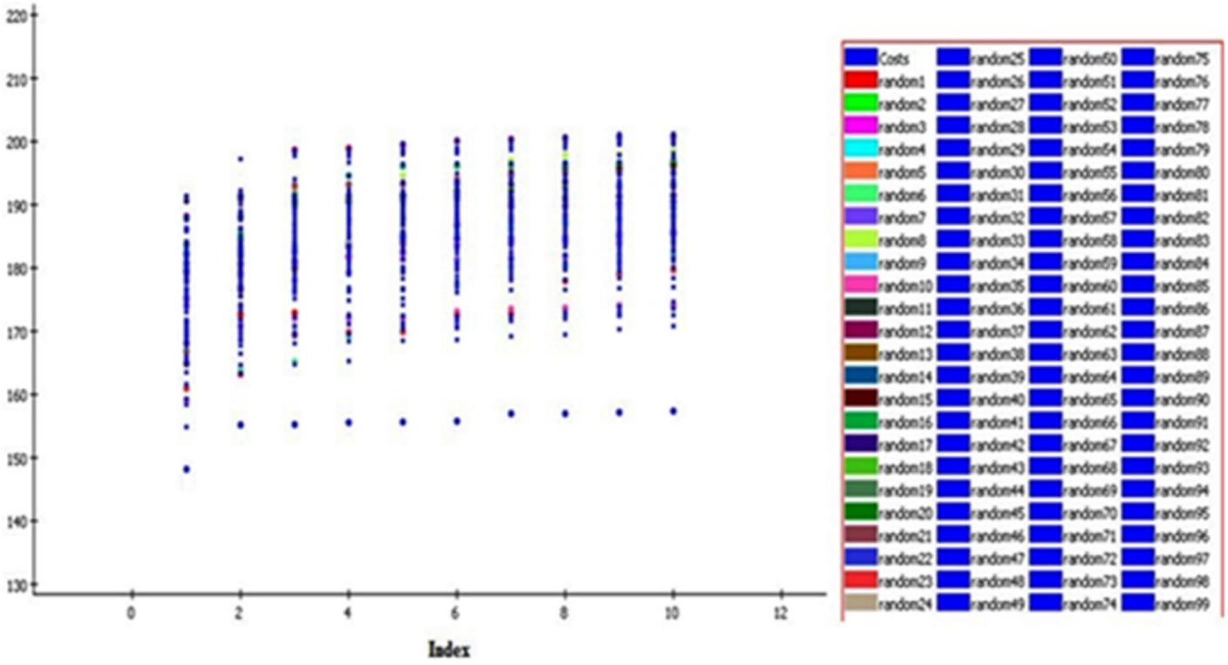

Figure S2. Plot of 99% Fischer's randomization test of pharmacophore model 1.
